# An Effective Interface Tracking Method for Simulating the Extrudate Swell Phenomenon

**DOI:** 10.3390/polym13081305

**Published:** 2021-04-16

**Authors:** Ahmad Fakhari, Željko Tukovic, Olga Sousa Carneiro, Célio Fernandes

**Affiliations:** 1 Institute for Polymers and Composites, Department of Polymer Engineering, Campus of Azurém, University of Minho, 4800-058 Guimarães, Portugal; ahmadfakhari@gmail.com (A.F.); olgasc@dep.uminho.pt (O.S.C.); cbpf@dep.uminho.pt (C.F.); 2 Faculty of Mechanical Engineering and Naval Architecture, University of Zagreb, Ivana Lučića 5, 10000 Zagreb, Croatia

**Keywords:** extrusion, extrudate swell, interface tracking, least-squares volume-to-point interpolation, consistent PISO, finite volume method, OpenFOAM

## Abstract

The extrudate swell, i.e., the geometrical modifications that take place when the flowing material leaves the confined flow inside a channel and moves freely without the restrictions that are promoted by the walls, is a relevant phenomenon in several polymer processing techniques. For instance, in profile extrusion, the extrudate cross-section is subjected to a number of distortions that are motivated by the swell, which are very difficult to anticipate, especially for complex geometries. As happens in many industrial processes, numerical modelling might provide useful information to support design tasks, i.e., to allow for identifying the best strategy to compensate the changes promoted by the extrudate swell. This study reports the development of an improved interface tracking algorithm that employs the least-squares volume-to-point interpolation method for the grid movement. The formulation is enriched further with the consistent second-order time-accurate non-iterative Pressure-Implicit with Splitting of Operators (PISO) algorithm, which allows for efficiently simulating free-surface flows. The accuracy and robustness of the proposed solver is illustrated through the simulation of the steady planar and asymmetric extrudate swell flows of Newtonian fluids. The role of inertia on the extrudate swell is studied, and the results that are obtained with the newly improved solver show good agreement with reference data that are found in the scientific literature.

## 1. Introduction

Free-surface flows are encountered in many polymer processing and environmental applications [1,2]. Nevertheless, the variety of the analytical solutions for free-surface flows is usually very limited, even for very simple cases [3]. On the other hand, the experimental observations of real phenomena are onerous [3], and many experimental techniques are suitable for single-phase flows and undergo many difficulties to be extended to two-phase flows [4]. For these reasons, the use of numerical simulations to provide useful information about free-surface flows would be of great advantage. Flows with a free-surface are difficult to be modeled since the free-surface is a moving boundary, whose location is merely known initially, and it has to be determined later during the simulation [5]. There are different ways of modeling free-surface flows: the Interface Tracking (IT) approach, in which the free-surface is tracked using a sharp interface, and a dynamic computational grid is applied to follow the movement of the free-surface; and, the Interface Capturing (IC) approach, in which the free-surface is not treated as a sharp interface and, generally, the computational grid is static. Among the methods following the IC approach, the Marker-And-Cell (MAC) [6] method is based on a finite difference scheme applied to an Eulerian grid to solve the Navier–Stokes equations for the fluid flow motion, and resort to Lagrangian virtual particles to impose the movement at the free-surface, which is based on the velocity interpolated from the Eulerian grid [7,8]. Although the MAC method provides accurate information on the free-surface location [8,9], the computational cost is enormous, because the number of particles needed to reconstruct the free-surface is vast [5]. Instead of considering virtual Lagrangian particles to reconstruct the free-surface, the Volume-of-Fluid (VoF) method [10] solves a transport equation to calculate the volume fraction of each fluid present in the interface cells. Although the VoF method is more efficient than the MAC method, and is more practical for complex interface shapes, it considers the interface as a layer that usually covers one to three computational cells [5]; therefore, it does not provide an exact location of the free-surface. Despite many attempts to obtain the precise local curvature of the free-surface, when using the VoF method [9,11,12,13,14], this disadvantage still remains. One of the attempts employed to compute sharp interfaces for free-surface flows was implemented by Roenby et al. [15], by using the so-called isoAdvector algorithm, which follows two-steps: first, it computes an isosurface to evaluate the distribution of fluids inside the cells (known as the surface reconstruction step); and second, it advects the face–interface intersection line to obtain the time evolution within a time step of the submerged face area (known as the advection step). The method provided very satisfactory results in terms of volume conservation, boundedness, surface sharpness, and efficiency for two-dimensional and three-dimensional problems on both structured and unstructured meshes. The Level-Set (LS) method, which was proposed by Osher and Sethian [16], is another method that considers the contour of a smooth scalar function to specify the location of the free-surface. In this method, the value of the scalar function at a computational grid cell is often calculated based on the signed distance function [11,17,18]. Although the transition of fluid properties through the interface is smooth, it experiences difficulty if the curvature of the free-surface undergoes rapid changes [8]; and, also there is a need to define a transition region with a finite thickness [5]. Furthermore, mass conservation is an issue when using the LS method [5,8].

Because the VoF and LS methods are based on an implicit identification of the free-surface through the volume fraction and distance function, respectively, they are commonly called IC methods. From the reasoning that is explained above, the lack of prediction of the exact location of the free-surface, the high computational cost, and the precise calculation of the representation of surface forces (for example, surface tension) are the general disadvantages of the IC methods. On the other hand, IT methods use an explicit discretization of the interfacial discontinuity [19,20], which applies a body-fitted (boundary-fitted) grid and the free-surface boundary is tracked using mesh movement. Because the free-surface is treated as a sharp interface, it is the most accurate approach, albeit with limitations on the deformation of the free-surface.

Although limited in their application, IT methods have an important role in the numerical analyses of fundamental multiphase flows, such as the extrudate swell (or die swell) phenomenon exhibited by viscous fluids exiting long slits or capillary dies [21,22,23,24,25,26,27,28,29,30]. Extrudate swell, which is also known as Barus effect [31], occurs when melted polymer comes out of a die, where the size of the emerged polymer becomes different from the size of the die. This even happens in Newtonian fluids due to the streamline rearrangements at the die exit [32], which is around 13% for cylindrical channels and 19% for sheets at very low Reynolds numbers, while, at high Reynolds numbers, the swell shrinks and, finally, the Newtonian liquid comes out like a thinning jet. The extrudate swell of polymers is usually in a very low range of Reynolds numbers from $10^{-4}$ to $10^{-2}$, and the swell ratio can reach as high as 400% in specific cases, which are related to the viscoelastic character of polymers. Experiments have shown that, when the die length is short enough, the extrudate swell grows when compared to a case with the same mass flow rate, but a longer die, which is commonly attributed to the memory of entrance. Thus, the swell in short dies is a consequence of two components, the memory of entrance and also the normal stress release at the die exit. In addition to these parameters, temperature also influences extrudate swell. The thermal effects can increase the extrudate swell up to 15%. If the die temperature is lower than the melted polymer, the viscosity of the melted polymer increases at the wall and, therefore, the flow becomes limited and it undergoes a lower swell when compared to the case where the die temperature is higher than the melted polymer, where the liquid is less viscous at the wall, which lubricates the flow and results in a larger swell. Another important parameter for the polymer extrudate swell is the molecular structure of the polymer, since the first normal stress difference that appears at the die exit is affected by the molecular weight distribution. Furthermore, long chain branching enhances extrudate swell in polymers [33].

It is crucial to know the dimension of the emerging polymer from the die, since it determines the exact size of the extruded products; therefore, many attempts have been made to obtain equations for anticipating the swell ratio [31,34]. As an example, Tanner [35] introduced an equation for the swell ratio of a Maxwell-type constitutive equation of a viscoelastic fluid, and then revised it several years later [36] while considering that the first normal stress difference obeys $N_{1}=k\tau^{m}$, where τ is the fluid shear stress and *k* and *m* are model parameters, instead of the initial assumption $N_{1}=\mathrm{constant}\times \tau^{2}$. A large number of investigations of the die swell phenomenon are reported in the literature, which goes from two-dimensional (2D) to three-dimensional (3D) flows, from Newtonian to viscoelastic fluids rheology or from isothermal to non-isothermal flows. Crochet and Keunings [21] presented a 2D numerical investigation on slit die swell with three finite element meshes and two different techniques, a mixed and an extended u−v−p methods, and concluded that the swelling ratio depends upon the method that is used for its calculation and, for a given method, it is highly dependent upon mesh refinement. Subsequently, Mitsoulis et al. [22] used viscometric flow equations to simulate the swelling of viscoelastic fluids from long slit and capillary dies. The obtained results showed that, even when using this simple model for viscoelastic calculations, they were in good qualitative agreement with other numerical simulations, which employ a constitutive equation satisfying tensorial invariance, but accelerate the breakdown of the iterative scheme. Another work from the group of J. Vlachopoulos was presented by Karagiannis et al. [23], which studied the 3D free surface die swell of a Newtonian fluid in different geometries, specifically square, rectangular, equilateral triangular, bow-tie, and key-hole-shaped geometries. The obtained results were compared with experimental measurements and other numerical calculations with favourable agreement. The swelling ratios were found to strongly depend on the die geometry. The group of Crouchet also presented calculations for steady state 3D free surfaces of Newtonian and power-law fluids in the work of Wambersie and Crochet [24]. They combined a pseudo-transient marching technique, a decoupling algorithm, and a conjugate gradient solver to reduce the cost of the 3D calculations. The method was employed to study the circular, square, and rectangular die-swell problems, where the effects of inertia and shear-thinning were revealed. The works of Legat and Marchal [25,26] addressed the prediction of 3D free surface extrudate flows with a fully implicit finite element algorithm, in the sense that a Newton–Raphson scheme was applied to all variables and is geometrically general. The algorithm was employed to compute the extrudate swell of a rectangular die and in various complex sections containing multiple corners. The obtained results showed that the extrudate shape exhibits large deformations in the vicinity of all re-entrant corners, which would not be possible to predict in 2D simulations. Subsequently, the works of Georgiou and Boudouvis [27] and Mitsoulis et al. [28] studied the effects of inertia, surface tension, gravity, slip, and compressibility for both the 2D planar and axisymmetric extrudate-swell flows of Newtonian fluids. Recently, 3D isothermal and non-isothermal viscoelastic flow calculations with a transient finite element method for predicting extrudate swell of domains containing sharp edges were conducted by Spanjaards et al. [29,30]. The obtained results showed that the extrudate swelling is highly dependent on the rheological parameters and the constitutive model used, and that the wall temperature of the die can lead to a change in the bending direction. All of the presented works employed the finite element method to solve the problem of the extrudate swell for Newtonian and viscoelastic fluids. The present work aims to revisit the Newtonian extrudate swell flow problem by using the finite volume method, which is the core of the open-source computational library OpenFOAM [37].

When dealing with a steady state process, which is the case of profile extrusion, IT is usually the best alternative, since it does not present the problems that are related to interface diffusion inherent to the IC methods. OpenFOAM [37] comprises a solver to simulate free-surface flows following an IT approach, which was proposed by Tuković and Jasak [38]. One of the disadvantages generally raised to the IT methods is their computational efficiency, and many attempts have been carried out in order to increase the convergence rate of the pressure–velocity calculations [39,40,41]. Tuković et al. [41] proposed a non-iterative Pressure-Implicit with Splitting of Operators (PISO) algorithm based on extrapolation of mass flux, nodal velocity, and pressure from two previous time steps in order to have an approximation of these quantities in the new time step, and obtained a second order temporal accuracy in the cases with static and dynamic meshes.

This work aims to assess the capability of the solver that was developed by Tuković and Jasak [38] with the non-iterative PISO algorithm proposed by Tuković et al. [41] to efficiently simulate the extrudate swell phenomenon. For this purpose, the developed solver couples the *interfaceTrackingFvMesh* and *interTrackMeshMotion* libraries that are available in OpenFOAM [37] with the consistent second-order time-accurate non-iterative PISO algorithm. A least-squares volume-to-point interpolation method for the grid movement, which enables an efficient and accurate tracking of the free-surface motion, is employed. The enhanced algorithm is used to simulate the steady-state Newtonian extrudate swell problem in both planar and axisymmetric geometries for a parametric study of the effects of inertia, and the obtained results are compared with the reference data of Mitsoulis et al. [28]. Notice that, although the results presented here are limited to Newtonian fluids, the effects which are discussed can be qualitatively applied to all fluids (e.g., viscoelastic fluids). The main aim of this work is to present an open source finite volume numerical framework that can handle the extrudate swell problem in an efficient way, which can be extended in the future to allow simulating other fluids rheology.

## 2. Mathematical Formulation

In this section, the governing equations and numerical method employed for simulating the two-phase fluid flow with a sharp interface are described, while using the finite-volume method and an interface tracking algorithm for the moving mesh. The numerical scheme developed in this work is enhanced with the consistent second-order time-accurate non-iterative PISO algorithm [39,40,41] to reduce the computational wall time of the simulations and, for the moving mesh calculation, a Laplacian scheme is used with a least-squares interpolation, which allows for robust and stable deformation of the interface.

### 2.1. Governing Equations

The mass and linear momentum conservation laws are the equations of motion governing the isothermal flow of incompressible Newtonian fluids inside an arbitrary volume *V* bounded by a closed surface *S*,

(1) $$\begin{array}{c} \oint_{S}n\cdot u\;dS=0, \end{array}$$

(2) $$\begin{array}{c} \frac{d}{dt}\int_{V}u\;dV+\oint_{S}n\cdot \left(u-u_{s}\right)u\;dS=\oint_{S}n\cdot \left(\nu \nabla u\right)\;dS-\int_{V}\nabla P\;dV, \end{array}$$

where n is the outward pointing unit normal vector on *S*, u is the fluid velocity, $u_{s}$ is the surface *S* velocity, ν is the fluid kinematic viscosity, and *P* is the kinematic pressure obtained by subtracting the hydrostatic kinematic pressure $P_{hydrostatic}=g\cdot r$ from the absolute pressure, where **g** is the gravitational acceleration and r is the position vector.

For an arbitrary moving volume, the relationship between the rate of change of the volume *V* and the velocity $u_{s}$ is defined by the geometrical(space)conservationlaw [42],

(3) $$\begin{array}{c} \frac{d}{dt}\int_{V}dV-\oint_{S}n\cdot u_{s}\;dS=0. \end{array}$$

When considering that the fluid phases are immiscible, the fluid flow Equations (1) and (2) can be used for each phase individually and, at the interface, the proper boundary conditions must be used.

#### 2.1.1. Kinematic Condition

**Figure 1:**
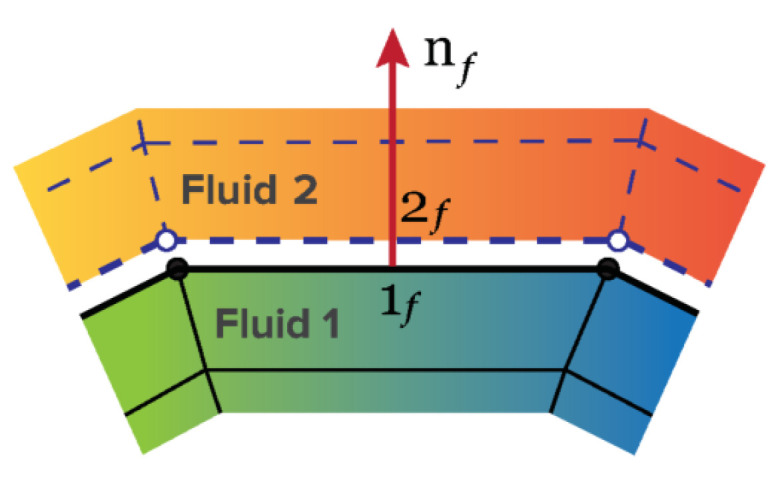
Representation of the interface with the mesh boundary faces.

The kinematiccondition states that the fluid velocities on the two sides of the interface, $u_{1f}$ and $u_{2f}$, must be continuous (see Figure 1),

(4) $$\begin{array}{c} u_{1f}=u_{2f}. \end{array}$$

#### 2.1.2. Dynamic Condition

From the momentum conservation law follows the dynamiccondition, which states that forces acting on the fluid at the interface are in equilibrium. The general form of the dynamiccondition at the interface, which gives the fundamental relationship between the jump in stress across an interface and the surface tension force, is given by,

(5) $$\begin{array}{c} \left[T_{2}-T_{1}\right]\cdot n=\nabla_{s}\sigma -\sigma \kappa n, \end{array}$$

where $T_{1}$ and $T_{2}$ are the stress tensors that are defined in terms of the local fluid pressure and velocity fields, as $T_{1}=-p_{1}I+\nu_{1}\left[\nabla u_{1}+{\left(\nabla u_{1}\right)}^{T}\right]$ and $T_{2}=-p_{2}I+\nu_{2}\left[\nabla u_{2}+{\left(\nabla u_{2}\right)}^{T}\right]$, respectively, σ is the interfacial tension and $\nabla_{s}=\left[I-\mathrm{nn}\right]\cdot \nabla =\nabla -n\frac{\partial }{\partial n}$ is the tangential gradient operator, which appears because σ and n are only defined on the surface. Equation (5) is a vectorial equation, which is often written in terms of its normal and tangential components. We proceed by deriving the normal and tangential force balances appropriately at a fluid–fluid interface that is characterized by an interfacial tension σ.

From the normal force balance follows the pressurejump across the interface [43],

(6) $$\begin{array}{c} p_{2}-p_{1}=\sigma \kappa -2\left(\nu_{2}-\nu_{1}\right)\nabla_{s}\cdot u, \end{array}$$

where $\kappa =-\nabla_{s}\cdot n$ is twice the mean curvature of the interface.

The tangential force balance yields a relation between the normal derivative of tangential velocity on the two sides of the interface [43],

(7) $$\begin{array}{c} \nu_{2}\left[n\cdot {\left(\nabla u_{t}\right)}_{2}\right]-\nu_{1}\left[n\cdot {\left(\nabla u_{t}\right)}_{1}\right]=-\nabla_{s}\sigma -n\left(\nu_{2}-\nu_{1}\right)\nabla_{s}\cdot u-\left(\nu_{2}-\nu_{1}\right)\left(\nabla_{s}u\right)\cdot n, \end{array}$$

where $u_{t}=\left(I-nn\right)\cdot u$ is the tangential velocity component.

### 2.2. Numerical Method

The numerical integration in time of the mathematical model that is described in Section 2.1 is performed using a second order accurate implicit method, and the integral form of the fluid flow equations are discretized in space using a second order accurate cell-centred unstructured finite volume method. Detailed information of the computational domain discretization, of the mathematical model, and interface tracking procedure can be found in Tuković and Jasak [38], being out of the scope of this work. Here, we devote our attention to the improvements performed in the numerical algorithm related to both efficiency and robustness of the calculations. For this purpose, the consistent non-iterative PISO algorithm [39,40,41] was employed to assure the pressure–velocity coupling in the calculation of the free surface flow studied in this work, and a least-squares volume-to-point interpolation method to compute the interface motion, were newly-implemented in the two-phase fluid-flow solver with a sharp interface.

#### 2.2.1. Consistent Non-Iterative PISO Algorithm

The rate of convergence of the collocated PISO algorithm is known to be problem dependent. In this segregated algorithm, a velocity correction term is neglected, which affects the path to convergence or may either cause the divergence of the numerical simulation, as a result of an exaggerated pressure correction field. Nevertheless, a common remedy for alleviating this problem is to under relax the pressure field. However, the rate of convergence remains a problem. In this work, we modified the original two-phase flow interface tracking solver, which is based in the segregated PISO algorithm, by approximating the velocity correction at the main grid point by a weighted average of the velocity corrections at the neighboring locations, the so-called consistent PISO algorithm [39,40,41].

A brief summary of the collocated PISO algorithm is described hereafter, along with the modification performed to the original formulation, by using the consistent counterpart of the algorithm, to improve the convergence rate of the calculations. A detailed analysis of both algorithms can be found in Van Doormaal et al. [39], Issa [40] and Tuković et al. [41]. First, the discretized momentum equations are given by

(8) $$\begin{array}{c} u_{C}+H_{C}\left[u\right]=-D_{C}^{u}{\left(\nabla P\right)}_{C}+B_{C}^{u}, \end{array}$$

with

(9) $$\begin{array}{c} H_{C}\left[u\right]=\sum_{f=NB(C)}\frac{a_{F}^{u}}{a_{C}^{u}}u_{F}, \end{array}$$

which is a weighted average that consists of the contribution of the neighbor cell with centroid *F*, $a_{F}^{u}$, and the contribution of the current cell with centroid *C*, $a_{C}^{u}$, to the velocity of the neighbour cells $u_{F}$. Notice that *f* refers to a face of the current cell, which shares it with a neighbor cell. The transient term contribution $D_{C}^{u}$ and the source term contribution $B_{C}^{u}$ are defined as the vector operators:

(10) $$\begin{array}{c} D_{C}^{u}=\frac{V_{C}}{a_{C}^{u}}, \end{array}$$

(11) $$\begin{array}{c} B_{C}^{u}=\frac{b_{C}^{u}}{a_{C}^{u}}, \end{array}$$

where $V_{C}$ is the volume of current cell *C*. Equation (8) is solved to obtain a momentum conserving velocity field $u^{*}$. Subsequently, the mass flow rate, ${\dot{m}}_{f}^{*}$, at the computational element faces should be updated using the Rhie–Chow [44] interpolation,

(12) $$\begin{array}{c} {\dot{m}}_{f}^{*}=u_{f}^{*}\cdot S_{f}=\bar{u_{f}^{*}}\cdot S_{f}-\bar{D_{f}^{u}}\left(\nabla P_{f}^{\left(n\right)}-\bar{\nabla P_{f}^{\left(n\right)}}\right)\cdot S_{f}, \end{array}$$

which allows for obtaining a momentum satisfying the mass flow rate ${\dot{m}}^{*}$. Here, $S_{f}$ denotes the normal face area vector and all of the values with an over bar are obtained by linear interpolation between the values at points *C* and *F*. Subsequently, we assemble the pressure correction equation [40],

(13) $$\begin{array}{c} \sum_{f=nb(C)}\left(\bar{D_{f}^{u}}{\left(\nabla P^{\prime }\right)}_{f}\cdot S_{f}\right)=-\sum_{f=nb(C)}{\dot{m}}_{f}^{*}+\sum_{f=nb(C)}\left(\bar{\sum_{F=NB(C)}\frac{a_{F}^{u}}{a_{C}^{u}}u_{F}^{{}^{\prime }}}\right)\cdot S_{f}, \end{array}$$

and solve it to obtain a pressure correction field $P^{\prime }$. In the original PISO algorithm, the last term in the RHS of Equation (13) is neglected, which affects the convergence rate, because, the larger this term, the higher the error present in the approximation at each iteration is. Finally, the mass flow rate at the element faces (${\dot{m}}_{f}^{**}$) and the pressure ($P_{C}^{*}$) and velocity ($u_{C}^{**}$) at the element centroids are updated with the corrected pressure field $P^{\prime }$ by,

(14) $$\begin{array}{c} {\dot{m}}_{f}^{**}={\dot{m}}_{f}^{*}+{\dot{m}}_{f}^{{}^{\prime }},\;{\dot{m}}_{f}^{{}^{\prime }}=-\bar{D_{f}^{u}}\nabla P_{f}^{{}^{\prime }}\cdot S_{f}, \end{array}$$

(15) $$\begin{array}{c} u_{C}^{**}=u_{C}^{*}+u_{C}^{{}^{\prime }},\;u_{C}^{{}^{\prime }}=-\bar{D_{C}^{u}}{\left(\nabla P^{{}^{\prime }}\right)}_{C}, \end{array}$$

(16) $$\begin{array}{c} P_{C}^{*}=P_{C}^{\left(n\right)}+\alpha^{p}P_{C}^{{}^{\prime }} \end{array}$$

where the superscript ${}^{\left(n\right)}$ denotes the solution at time t=n and $\alpha^{p}$ is the under relaxation factor for the pressure correction values, which increases the robustness and convergence behavior of the PISO algorithm.

Nevertheless, even when using under-relaxation factors in the PISO algorithm, the convergence rate is problem dependent. To improve the efficiency of the two-phase fluid flow calculations, we modified the original PISO algorithm by simply assuming that the velocity correction at point *C* is the weighted average of the corrections at the neighboring points,

(17) $$\begin{array}{c} u_{C}^{{}^{\prime }}\approx \frac{\sum_{F=NB(C)}a_{F}^{u}u_{F}^{{}^{\prime }}}{\sum_{F=NB(C)}a_{F}^{u}}\Rightarrow \sum_{F=NB(C)}a_{F}^{u}u_{F}^{{}^{\prime }}\approx u_{C}^{{}^{\prime }}\sum_{F=NB(C)}a_{F}^{u}, \end{array}$$

which can be written as,

(18) $$\begin{array}{c} \sum_{F=NB(C)}\frac{a_{F}^{u}u_{F}^{{}^{\prime }}}{a_{C}^{u}}\approx u_{C}^{{}^{\prime }}\sum_{F=NB(C)}\frac{a_{F}^{u}}{a_{C}^{u}}. \end{array}$$

Hence, the neglected term in the PISO algorithm (last term in the RHS of Equation (13)) is replaced by the approximate value that is given by Equation (18). Thus, in the consistent PISO algorithm a smaller term is discarded, which allows for obtaining more accurate velocity corrections with the momentum equations. Therefore, the convergence rate of the consistent PISO algorithm is higher than the one of the original PISO algorithm. Notice that our approach to simulate steady state free surfaces is, in fact, a time-dependent marching technique that allows separately calculating the free surface movement and the other fields at the different time steps, which reduces the number of iterations needed to converge to a steady solution [24].

A detailed analysis regarding the numerical setup for the PISO algorithm allowed for concluding that 10 outer corrector loops and three pressure–velocity correctors were needed to obtain stable and converged iterative solutions for all the cases simulated in this work. Additionally, for the PISO algorithm, the simulations only converged at maximum with a Courant number of 0.2, while using the consistent PISO algorithm the simulations were performed with a Courant number of 1.

#### 2.2.2. Least-Squares Volume-to-Point Interpolation

The mesh deformation is calculated using the Laplace mesh motion equation with variable diffusivity [45]. The method discretizes the motion equation using the cell-centred FV method, by which vertex displacements are obtained using a reconstruction procedure, instead of the commonly employed FE discretization. Detailed information regarding this procedure can be found in Tuković and Jasak [38] and Jasak and Tuković [45].

Following the work of Tuković et al. [46], we employ the weighted least-squares method and linear fitting function to reconstruct the vertex displacements from the cell-centre displacements of the cells surrounding the vertex, which increased the robustness of the free-surface flows calculation. It is worth noting that, without the least-squares volume-to-point interpolation, the original mesh motion algorithm available in OpenFOAM [37] was not able to compute the mesh deformation that occurs in the simulations of the extrudate swell flows presented in Section 3.

**Figure 2:**
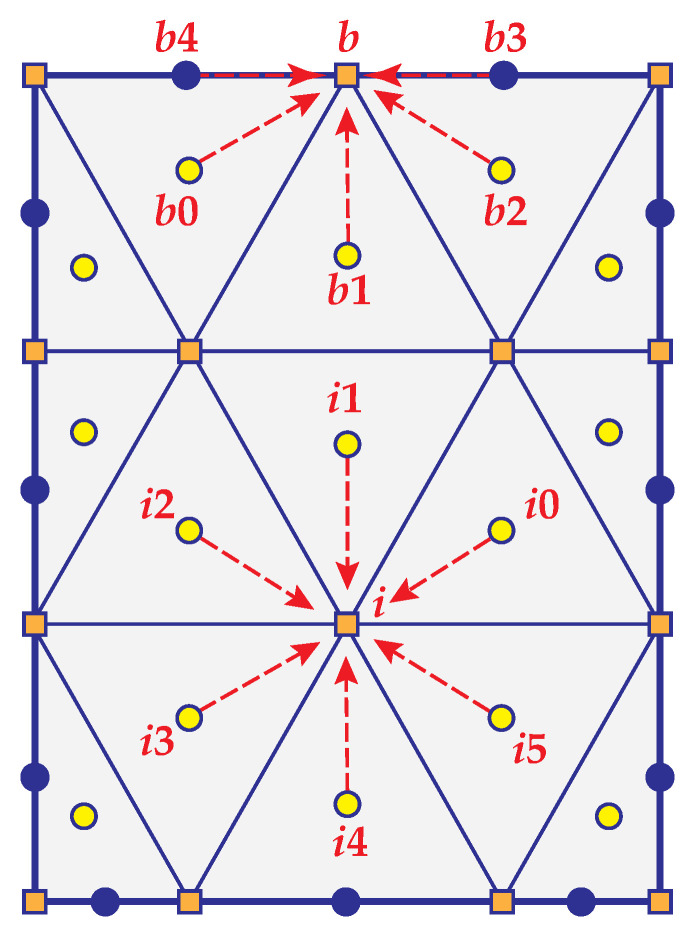
Finite volume mesh with an interpolation stencil for the vertex field value reconstruction. The interpolation stencil is given for the internal vertex *i* and boundary vertex *b*.

Consider the interpolation stencils for the internal (*i*) and the boundary (*b*) vertices that are given in Figure 2. The former is constituted of all cells sharing the vertex, while, in the latter, the boundary faces sharing the corresponding vertex are also included into the stencil. In the vicinity of each vertex *i* (or boundary *b*), a linear interpolation function is considered:

(19) $$\begin{array}{c} \phi \left(r\right)=\phi_{i0}+C_{i}\cdot \left(r-r_{i0}\right), \end{array}$$

where $C_{i}$ is the unknown coefficient vector, and the field value $\phi_{i0}$ and the reference position $r_{i0}$ are obtained as the weighted average of cell-centre field and positions values, respectively:

(20) $$\begin{array}{c} \phi_{i0}=\frac{\sum_{j=1}^{n}w_{ij}\phi_{ij}}{\sum_{j=1}^{n}w_{ij}}, \end{array}$$

(21) $$\begin{array}{c} r_{i0}=\frac{\sum_{j=1}^{n}w_{ij}r_{ij}}{\sum_{j=1}^{n}w_{ij}}, \end{array}$$

where $\phi_{ij}$ is the field value in the centre of cell *j* in the interpolation stencil of the vertex *i*, $r_{ij}$ is the centre of cell *j* in the interpolation stencil of the vertex *i*, and $w_{ij}$ is the weighting factor calculated as the inverse square distance between the position of vertex *i* and the centre of cell *j*. Finally, to obtain the unknown coefficient vector, $C_{i}$, the weighted least-squares method is employed:

(22) $$\begin{array}{c} C_{i}=\left[{\left(X^{T}WX\right)}^{-1}X^{T}W\right]\cdot \Phi_{i}, \end{array}$$

where X is the n×3 matrix whose row *j* is the position vector $\left(r_{ij}-r_{i0}\right)$ of the cell *j* in the interpolation stencil of the vertex *i*, W is the diagonal matrix whose elements are the weighting factors for all cells in the interpolation stencil of the vertex *i*, and $\Phi_{i}$ is the vector constituted by the elements $\left(\phi_{ij}-\phi_{i0}\right)$ for all cells in the interpolation stencil of the vertex *i*.

## 3. Results and Discussion

### 3.1. Planar Extrudate Swell of Newtonian Fluids

**Figure 3:**
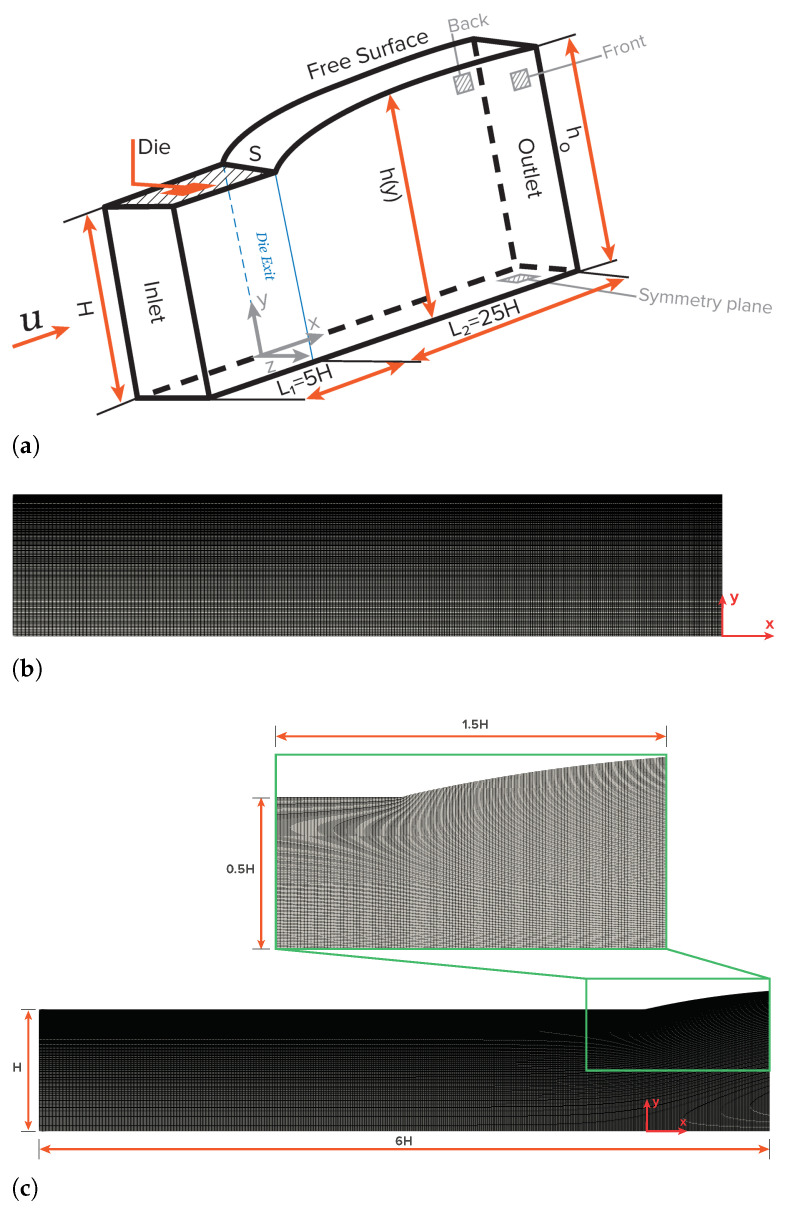
Schematic representation of the planar extrudate swell domain geometry and boundary faces (**a**), of an indicative discretization mesh at the initial time-step t=0 (**b**), and at steady-state (**c**).

The first benchmark case study that will be discussed is the planar extrudate swell of Newtonian fluids. Figure 3 shows a schematic representation of the computational flow domain, the boundary faces, and an indicative discretization mesh for the initial time-step (t=0) and at steady-state. Cartesian coordinates are employed for the description of the planar flow domain, thus x=(x,y). The half width of the planar channel is denoted as *H*, which is considered as the scaling length. The inlet plane is taken sufficiently far upstream from the exit so that the flow is fully developed with a mean velocity *U*. Along the axis of symmetry the standard symmetry conditions are imposed. At the solid die wall the no-slip (tangential velocity is zero) and no penetration (normal velocity is zero) conditions are imposed. At the free-surface the kinematic, Equation (4), and dynamic conditions, Equations (6) and (7), are imposed. Finally, the outflow plane is taken sufficiently far downstream from the exit, so that the flow is uniform. The die exit of the planar domain is located at x=5H from the inlet, and the outflow is located at x=25H from the die exit.

In this section, we compare the results that were obtained with the newly-improved interface tracking algorithm with those given by Tanner [1], Georgiou and Boudouvis [27] and Mitsoulis et al. [28]. First, a mesh convergence sensitivity analysis is performed. Table 1 summarizes the main characteristics of the meshes employed in this preliminary study. The rectangular domain was initially discretized with 320 cells in the streamwise (x), 37 cells in wall normal (y), and one cell in span-wise directions, with this mesh being named M1. A linear stretch was employed in streamwise and wall normal directions to have the highest resolution at downstream edge of the die (at S) with the largest to smallest cell aspect ratio being equal to 2.5. An extensive investigation was carried out on the necessary grid spacing aspect ratio, and it was concluded that this value was efficient enough to track the free-surface. Five different meshes, designated M1, M2, M3, M4, and M5, were found to be sufficient to obtain accurate results for this problem. Table 1 shows the number of mesh elements used in each direction of the computational domain, the total number of mesh elements, and the degree of freedom (dof) of the numerical algorithm for each level of mesh refinement. Notice that each mesh is obtained from the preceding one by multiplying the number of cells in each direction by a factor of 1.5.

First, we compare our mesh convergence predictions of the steady-state swell ratio, χ, with the results found in the scientific literature [1,27,28]. The swell ratio is defined as the height of the free-surface away from the die exit, where the plug flow has been established, divided by the die height, i.e., $\chi =h_{0}/H$. For the mesh convergence analysis, we employed the Newtonian fluid at creeping flow conditions (Re=0.1). Table 1 shows the extrudate swell ratio for the different mesh resolutions. The accuracy of the developed code is estimated via the application of Richardson’s extrapolation [47] to the limit, by using the three most refined levels of mesh discretization. The extrapolated value of the swell ratio is equal to χ=1.187, similar to the reference data of Mitsoulis et al. [28], which reported a value of χ=1.186 using a FEM numerical algorithm, to the estimated extrapolated value of Tanner [1] with χ=1.190±0.002, and to the converged results obtained by Georgiou and Boudouvis [27] of χ=1.186, as shown in Table 1. Additionally, notice that the relative error calculated between the extrudate swell ratio obtained using M5 (the most refined mesh) and the one extrapolated using the Richardson’s technique [47] is equal to $\left(\chi_{M5}-\chi_{Extrapolated}\right)/\chi_{Extrapolated}\times 100\approx 0.7\%$. Additionally, notice that, even when using M1 (the coarsest mesh), the relative error is only 2.3%. In terms of computational wall time, the simulation of the planar extrudate swell with M1 and M5 took approximately 0.94 and 24.3 hours, respectively, where all of the computations were performed in parallel using 80 processors on a computer with a 2.70 GHz Intel Xeon CPU E5-2680.

**Figure 4:**
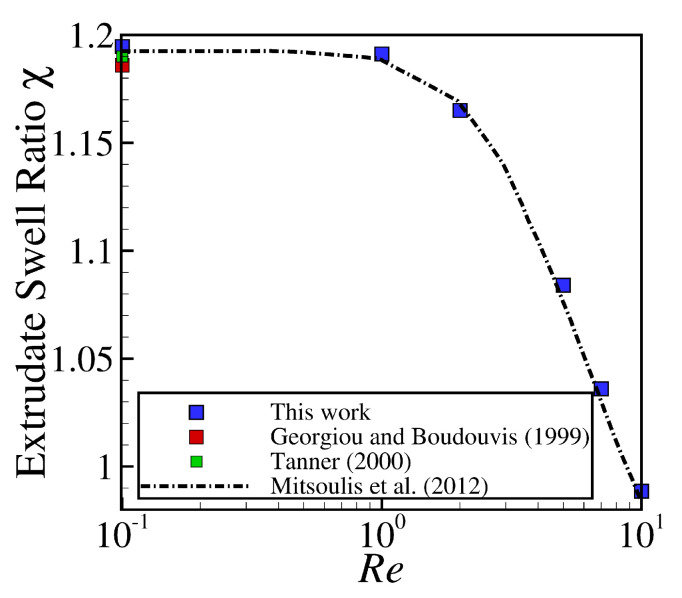
Steady state extrudate swell ratio χ for the simulations using the planar extrudate swell domain geometry of Newtonian fluids at Re={0.1,1,2,5,7,10}. Dashed lines, and red and green square symbols represent the results obtained by Mitsoulis et al. [28], Georgiou and Boudouvis [27] and Tanner [1], respectively, and the blue square symbols represent the results obtained by the newly-improved interface tracking code.

Subsequently, calculations for studying the robustness of the newly-improved interface tracking algorithm were pursued, by increasing the Reynolds number (Re≤10) in the planar extrudate swell domain geometry. Figure 4 depicts the extrudate swell ratio, χ, as a function of the Re number, where the blue square symbols are the results obtained by the newly-improved interface tracking code using M5, and the dashed lines and red and green square symbols are the results found in the scientific literature [1,27,28]. As can be seen, the numerical results obtained are in very good agreement with the reference data.

**Figure 5:**
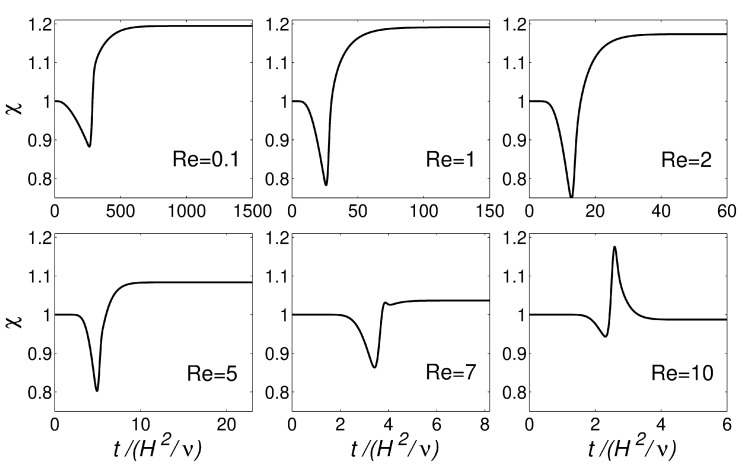
Transient evolution of extrudate swell ratio (χ) with dimensionless time ($t/\left(H^{2}/\nu \right)$ for the simulation of the planar extrudate swell domain geometry of Newtonian fluids at Re={0.1,1,2,5,7,10}.

Figure 5 shows the transient evolution of χ for the planar extrudate swell domain geometry at Re={0.1,1,2,5,7,10}. The numerical results obtained for Re<7 show an undershoot in the values of χ, before reaching the steady-state value. For Re≥7, after the undershoot in the values of χ, an overshoot is present, and, ultimately, the steady-state value is reached, being approximately 0.99. This increase of inertia allowed for verifying the robustness of the improved interface-tracking algorithm, namely the Laplacian solver with least-squares volume-to-point interpolation, to handle abrupt changes in the mesh motion.

**Figure 6:**
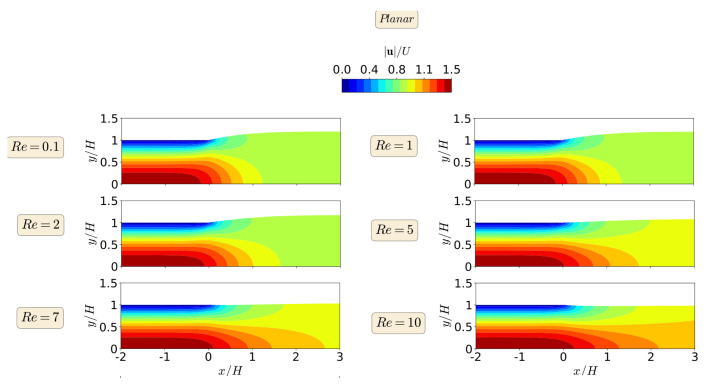
Steady state velocity vector magnitude contours for the planar extrudate swell flow of Newtonian fluids, at Re=0.1 (**top**), 1, 2, 5, 7, and Re=10 (**bottom**).

**Figure 7:**
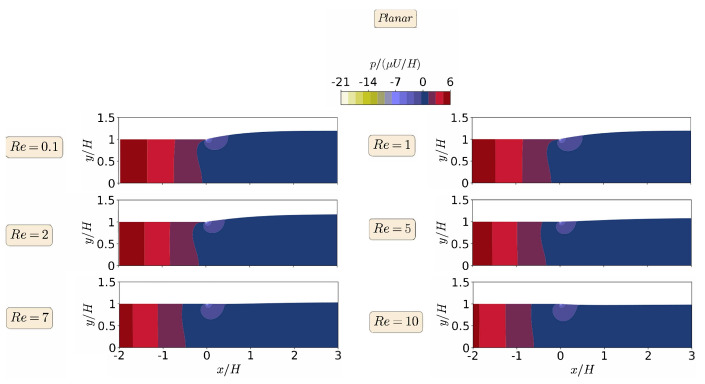
Steady state pressure contours for the planar extrudate swell flow of Newtonian fluids, at Re=0.1 (**top**), 1, 2, 5, 7, and Re=10 (**bottom**).

The steady-state results for the primary field variables, magnitude of the velocity vector |u|, and pressure field *p*, in the form of contours, are shown in Figure 6 and Figure 7, respectively, for the planar extrudate swell domain geometry, and at different Re numbers. From a detailed inspection of Figure 6 and Figure 7, we can see that inertia substantially reduces the free-surface height, as already shown in Figure 4. This fact occurs, because inertia pushes the material to the center of the domain, generating a peak of negative pressure in the upper point near the die exit. This result was expected to occur due to the Poiseuille flow that developed in the upstream region of the domain geometry [48]. Additionally, for Re=10, the contour of the magnitude of the velocity vector changes its behavior, when compared to the lower Re number cases. It can be seen that the maximum values of the velocity vector magnitude are extended after the die exit for the higher Re case. Finally, regarding the pressure contours, we can see that the increase of inertia does not change abruptly the pressure contours, and, at Re≥7, we see an extension of the minimum pressure values from the top corner of the die exit to the center of the channel, which seems to cause the reduction in the free-surface height.

Finally, the efficiency of the newly-improved interface-tracking solver was assessed by comparing the required CPU wall time per time-step when using the original PISO algorithm or the enhanced consistent-PISO algorithm. Table 2 shows a comparison of the dimensionless time-step $\Delta t/(H^{2}/\nu )$ employed in the simulations of the planar extrudate swell and the elapsed time per time-step that is required by both PISO and consistent PISO algorithms for Re={0.1,1,10}, while using mesh M5. For Re=0.1, the consistent-PISO algorithm allows using a time-step 21.4 times higher than the one that is used by the PISO algorithm, and the CPU wall time per time-step that elapsed using the consistent-PISO algorithm is approximately half of the one taken by the PISO algorithm. For Re=1 and Re=10, the scenario is also favorable to the consistent-PISO algorithm to the detriment of the PISO algorithm, which allows for concluding that the enhanced correction procedure for the p−U fields is advantageous for efficiently simulating the extrudate swell problem. Table 2 also shows the total calculation time in hours when using M5 and the consistent PISO algorithm. As can be seen, all of the calculations finished in less than 1.5 days.

### 3.2. Axisymmetric Extrudate Swell of Newtonian Fluids

**Figure 8:**
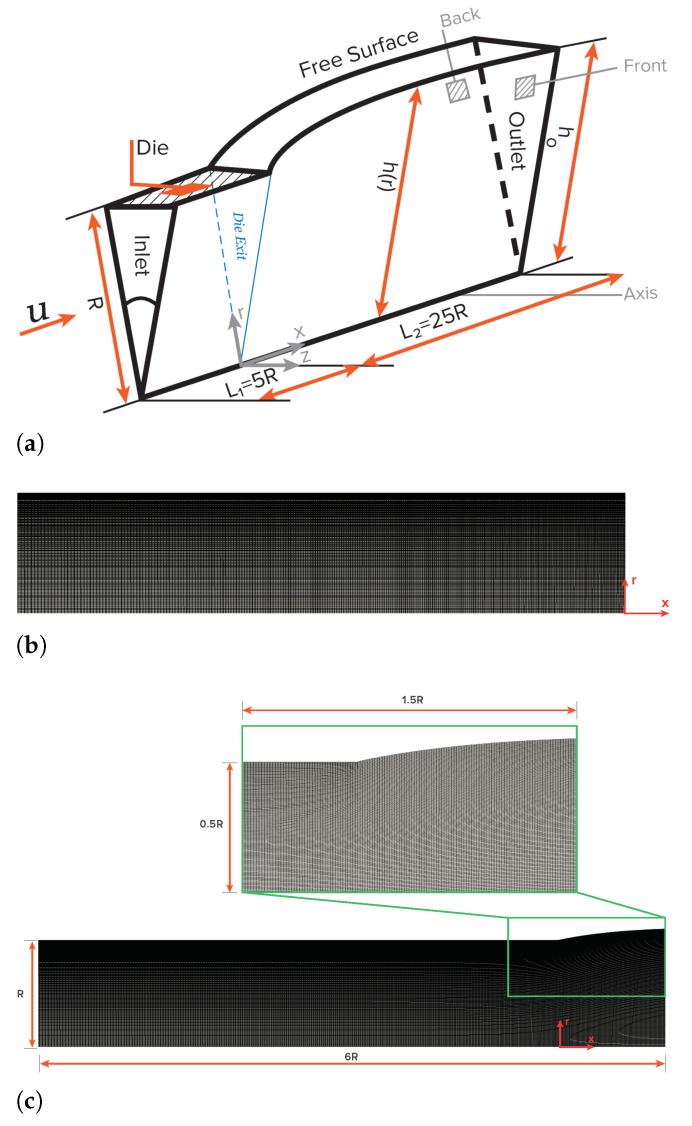
Schematic representation of the axisymmetric extrudate swell domain geometry and boundary faces (**a**), of an indicative discretization mesh at the initial time-step t=0 (**b**), and at steady-state (**c**).

The second benchmark case study that is discussed in this work is the axisymmetric extrudate swell of Newtonian fluids. Figure 8 shows a schematic representation of the computational flow domain, the boundary faces, and an indicative discretization mesh for the initial time-step (t=0) and at steady-state. Polar coordinates are employed for the description of the axisymmetric flow domain, thus x=(r,z). The half width of the axisymmetric channel is denoted as *R*, which is considered to be the scaling length. The boundary conditions imposed in the boundary faces are similar to the ones presented for the planar extrudate swell case study, with the exception that, in the axisymmetric domain, the two lateral boundary sides are considered to be wedge patches (i.e., the cylinder is specified as a wedge of small angle, e.g., 5° and one cell thick running along the plane of symmetry, straddling one of the coordinate planes), while, in the planar case study, they were considered as empty patches (i.e., this condition applies on each patch whose plane is normal to the third dimension for which no solution is required); and, instead of symmetryPlane at the bottom of the planar case, the axis of symmetry is considered as empty patch. The die exit of the axisymmetric domain is located at z=5R from the inlet, and the outflow is located at z=25R from the die exit.

**Figure 9:**
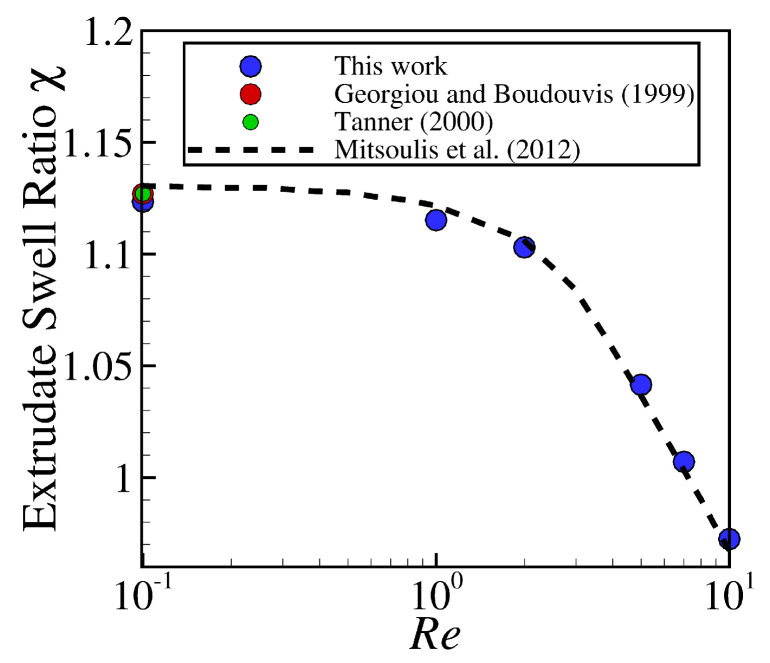
Steady state extrudate swell ratio χ for the simulations using the axisymmetric extrudate swell domain geometry of Newtonian fluids at Re={0.1,1,2,5,7,10}. Dashed lines, and red and green circle symbols represent the results that were obtained by Mitsoulis et al. [28], Georgiou and Boudouvis [27] and Tanner [1], respectively, and the blue circle symbols represent the results obtained by the newly-improved interface tracking code.

Figure 9 illustrates the extrudate swell ratio, χ, for different Re with mesh resolution M5 (see Table 1) using the axisymmetric domain geometry. Similarly to the planar domain geometry, generally, the extrudate swell ratio that is obtained by the newly-improved interface tracking solver using the axisymmetric domain is in good agreement with the reference data [1,27,28].

**Figure 10:**
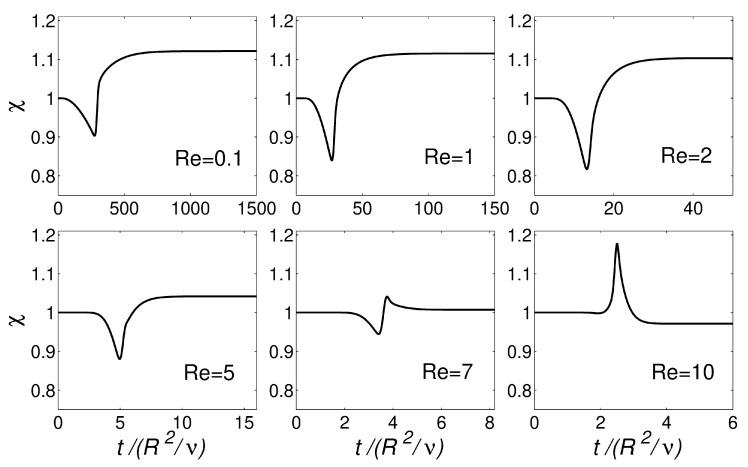
Transient evolution of extrudate swell ratio (χ) with dimensionless time ($t/\left(R^{2}/\nu \right)$ for the simulation of the axisymmetric extrudate swell domain geometry of Newtonian fluids at Re={0.1,1,2,5,7,10}.

Figure 10 shows the transient evolution of χ for the axisymmetric extrudate swell domain geometry at Re={0.1,1,10}. Again, the numerical results obtained for Re<7 show an undershoot in the values of χ, before reaching the steady-state value. When comparing with the planar case (see Figure 5), the magnitude of the undershoot is smaller for the present case, which can be attributed to the round boundary surface of the axisymmetric domain geometry. For Re≥7, an almost imperceptible undershoot is present when using the axisymmetric domain geometry (contrarily to the planar domain geometry), followed by an overshoot, and ultimately the steady-state χ value is reached, being approximately 0.97 (2% less than the value that is obtained for the planar domain geometry).

**Figure 11:**
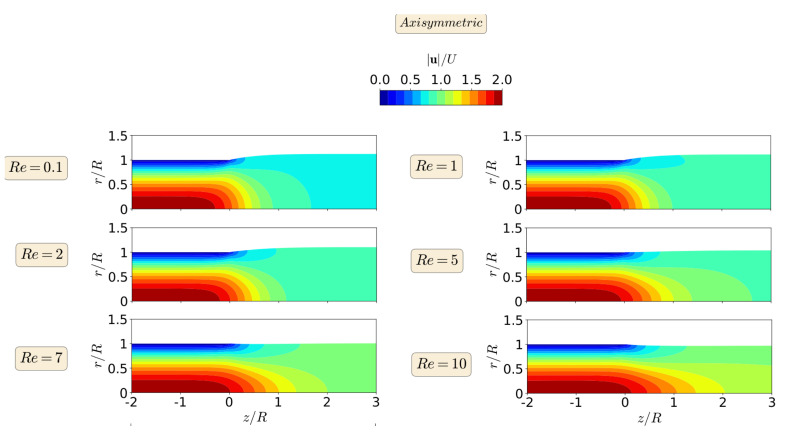
Steady state velocity magnitude contour for axisymmetric swell flow of Newtonian fluids, at Re=0.1 (**top**), 1, 2, 5, 7 and Re=10 (**bottom**).

**Figure 12:**
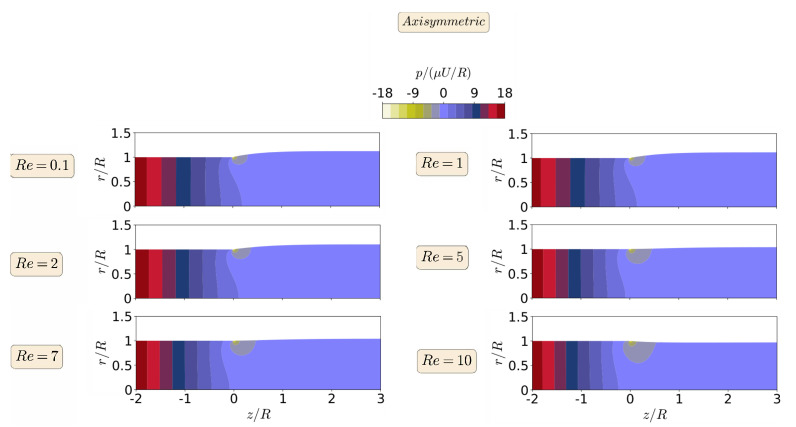
Steady state pressure contour for axisymmetric extrudate swell flow of Newtonian fluids, at Re=0.1 (**top**), 1, 2, 5, 7, and Re=10 (**bottom**).

The steady-state results for the primary field variables, magnitude of the velocity vector |u|, and pressure field *p*, in the form of contours are shown in Figure 11 and Figure 12, respectively, for the axisymmetric extrudate swell domain geometry and at different Re numbers. From a detailed inspection of Figure 11 and Figure 12, we can also see here that inertia substantially reduces the free-surface height, as already shown in Figure 9. Additionally, when compared to the lower Re number cases, we can see that, only for Re=10, the contour of the magnitude of the velocity vector changes its behavior, where the maximum values are extended until the die exit for the latter case. Again, the increase of inertia, does not change abruptly the pressure contours, and only at Re=10, we see an extension of the minimum pressure contours from the top corner of the die exit to the center of the channels, which seems to cause the reduction in the free-surface height (but with a lesser effect in the axisymmetric domain geometry).

When comparing the contour results from both planar and axisymmetric extrudate swell domain geometries, we can state that the maximum velocity vector magnitude is obtained for the axisymmetric domain, being two times the magnitude of the inflow average velocity, in contrast with the planar case, where it is only 1.5 times higher than the magnitude of the inflow average velocity. This result was expected to occur due to the Poiseuille flow, which is developed in the upstream channel of both domains [48]. Finally, regarding the pressure contours, we can see that, for the axisymmetric domain, the computed maximum and minimum pressure values are symmetric, as expected, due to domain symmetry, in contrast with the planar domain case, where the minimum pressure value that is obtained is 3.5 times lesser than the maximum one. In both planar and axisymmetric cases, as the Re increases the extrudate swell ratio and pressure decreases, which physically states that the inertia forces stretch out the material and prevent the swelling, with an immediate effect on the reduction of pressure losses [28].

Finally, Table 3 shows a comparison of the dimensionless time-step $\Delta t/(H^{2}/\nu )$ employed in the simulations of the axisymmetric extrudate swell and the CPU wall time (s) per time-step required by both PISO and consistent PISO algorithms for Re={0.1,1,10} using mesh M5. For Re=0.1, the consistent-PISO algorithm allows for using a time-step 21.0 times higher than the one used by the PISO algorithm, and the CPU wall time per time-step elapsed using the consistent-PISO algorithm is 0.58 times the one taken by the PISO algorithm. Again, for Re=1 and Re=10, the scenario is also favorable to the consistent-PISO algorithm in detriment of PISO algorithm. Additionally, notice that, when comparing with the values that are shown in Table 2, the ratios of CPU wall time per time-step (C-PISO/PISO) are lower for the planar extrudate swell, which mean that the CPU wall time gains using the C-PISO algorithm seems to be higher for non-smooth geometries. Table 2 also shows the total calculation time in hours when using M5 and the consistent PISO algorithm. As for the planar extrudate swell, all of the calculations finished in less than 1.5 days.

## 4. Conclusions

A numerical formulation for efficient moving mesh interface tracking simulations of free-surface flows was presented and implemented using the finite-volume method. The implementation was performed in the open-source OpenFOAM framework [37], where the interface is tracked in a semi-implicit manner inside the consistent second-order time-accurate non-iterative Pressure-Implicit with Splitting of Operators (consistent PISO) algorithm for the numerical solution of incompressible fluid flows. Additionally, the moving mesh was adjusted to the time varying shape of the interface, using a Laplacian scheme with a least-squares volume-to-point interpolation, which allowed for robust and stable deformations of the interface.

The improved algorithm was assessed in terms of the accuracy and efficiency for the fluid flow simulations in planar and axisymmetric extrudate swell of Newtonian fluids. A mesh sensitivity analysis allowed for obtaining a grid refinement level from which the calculated extrudate swell ratio differs from the extrapolated value of 0.7%. Subsequently, the robustness of the numerical algorithm was pursued, increasing the Reynolds number from Re=0.1 to Re=10. The extrudate swell ratio that was obtained for both domains compared well with the results found in the scientific literature for that range of Re. Additionally, the contours for the magnitude of the velocity vector and pressure fields are also shown, and a detailed study of the contours reveals that the obtained results are physically meaningful. Finally, the efficiency of the improved numerical solver was evaluated by comparing the CPU wall time (s) per time-step for both the PISO and consistent PISO algorithms. The results obtained allowed for concluding that the consistent-PISO is, at maximum, 47% and 42% faster than the PISO algorithm for the planar and axisymmetric extrudate swell domain geometries, respectively.

In summary, the results presented here show that the newly-improved interface tracking code, developed using an open-source framework, can accurately and efficiently predict the Newtonian extrudate swell. The code that was implemented here is being currently extended to handle viscoelastic fluid flow calculations and non-isothermal processes. For the viscoelastic fluid flow calculations, we will use the quasi-linear Oldroyd-B and exponential PTT rheological models. The former will be used due to the numerical instabilities that are caused by the infinite polymeric stresses generated at singular points, which will verify the robustness of the numerical implementation; and the latter will be used because it is more suitable for approximating the behavior of polymer melts, where the extensibility parameter introduces elongational and shear thinning in the fluid model. Additionally, the solvent and polymeric viscosities and the relaxation time of the viscoelastic fluid will be considered as temperature dependent, by employing the Williams–Landel–Ferry relation.

## Figures and Tables

**Table 1:** Finite volume mesh characteristics used in the simulations and Newtonian base results for the extrudate swell ratio, χ, at Re=0.1 while using the planar extrudate swell domain geometry.

Mesh	$N_{x}\times N_{y}$	No. of Elements	No. of Dof	χ	Error (%)
M1	320×37	11,840	59,200	1.214	2.3
M2	480×56	26,880	134,400	1.207	1.7
M3	720×84	60,480	302,400	1.201	1.2
M4	1080×126	136,080	680,400	1.198	0.9
M5	1620×189	306,180	1,530,900	1.195	0.7
Extrapolated	-	-	-	1.187
Mitsoulis et al. [28]	-	-	-	1.186
Tanner [1]	-	-	-	1.190 ± 0.002
Georgiou and Boudouvis [27]	-	-	-	1.186

**Table 2:** Comparison of the dimensionless time-step $\Delta t/(H^{2}/\nu )$ employed in the simulations and the CPU wall time (s) per time-step required by the PISO and consistent PISO algorithms for all Re, using mesh M5, for the planar extrudate swell flow of Newtonian fluids.

Re	$\Delta t/(H^{2}/\nu )$			CPU Wall Time (s) Per Time-Step			
	C-PISO	PISO	C-PISO/PISO	C-PISO	PISO	C-PISO/PISO	Total calculation time [h]
0.1	0.0685	0.0032	21.4	8	15	0.53	24.3
1	0.0069	0.0014	4.9	8	11	0.72	32.2
10	0.0007	0.0001	7.0	7	11	0.63	13.9

**Table 3:** A comparison of the dimensionless time-step $\Delta t/(R^{2}/\nu )$ employed in the simulations and the CPU wall time (s) per time-step required by the PISO and consistent PISO algorithms for all Re, using mesh M5, for the axisymmetric extrudate swell of Newtonian fluids.

Re	$\Delta t/(R^{2}/\nu )$			CPU Wall Time (s) Per Time-Step			
	C-PISO	PISO	C-PISO/PISO	C-PISO	PISO	C-PISO/PISO	Total calculation time [h]
0.1	0.0652	0.0031	21.0	7	12	0.58	22.4
1	0.0064	0.0013	4.9	7	9	0.78	30.4
10	0.0006	0.0001	6	5	9	0.56	11.6

## Data Availability

Not applicable.
